# Imbalanced intracellular nutrient stoichiometries drive the regional structural variation of microeukaryotic communities in paddy fields

**DOI:** 10.1093/ismeco/ycae119

**Published:** 2024-10-10

**Authors:** Pengfei Sun, Eleonora Silvano, Yin Chen, Yonghong Wu

**Affiliations:** State Key Laboratory of Soil and Sustainable Agriculture, Institute of Soil Science, Chinese Academy of Sciences, 71 East Beijing Road, Nanjing 210008, China; School of Life Sciences, University of Warwick, Coventry CV4 7AL, United Kingdom; University of Chinese Academy of Sciences, No.188, Tianquan Road, Nanjing 211135, China; School of Life Sciences, University of Warwick, Coventry CV4 7AL, United Kingdom; School of Biosciences, The University of Birmingham, Edgbaston, Birmingham B15 2TT, United Kingdom; State Key Laboratory of Soil and Sustainable Agriculture, Institute of Soil Science, Chinese Academy of Sciences, 71 East Beijing Road, Nanjing 210008, China; University of Chinese Academy of Sciences, No.188, Tianquan Road, Nanjing 211135, China

**Keywords:** microeukaryotic community, intracellular nutrient stoichiometry, local species richness, regional structural variations, community assembly

## Abstract

Periphytons serve as critical microbial nutrient sinks at the soil–water interface, influencing biogeochemical cycles and nutrient migration in paddy fields. Despite their importance, the impact of accumulated intracellular nutrients on the spatial dynamics and community assembly of periphytons, particularly their microeukaryote communities, remains unclear. To address this gap, we examined the nutrient accumulation potential and its effects on microeukaryotes in periphytons from 220 paddy fields spanning up to 3469 km across three temperature zones. Our study reveals that the periphytons exhibit varying capacities to accumulate carbon, nitrogen, and phosphorus, leading to imbalanced intracellular nutrient stoichiometries (carbon-to-nitrogen ratio = 10.3 ± 2.1, carbon-to-phosphorus ratio = 30.9 ± 13.1, nitrogen-to-phosphorus ratio = 3.1 ± 1.3). This stoichiometric imbalance induces intracellular environmental heterogeneity, which partially influences the local species richness of microeukaryotic communities and their regional structural variations on a large scale. Contrary to the typical latitudinal diversity gradient theory, local microeukaryotic species richness follows a distance-decay model, with both deterministic and stochastic processes contributing to community assembly. These results underscore the complex interplay of environmental filtering, species interactions, and dispersal dynamics in shaping the structure and adaptability of microeukaryotic communities within periphytons. This study contributes to a broader understanding of the factors driving regional structural variations of microeukaryotes at the soil–water interface in agricultural landscapes.

## Introduction

Both prokaryotes and microeukaryotes in agroecosystems play diverse and essential roles, including driving nutrient cycling, shaping soil fertility, and influencing crop productivity [1–3]. This underscores the need to give equal attention to both eukaryotic and prokaryotic microorganisms. However, research on microeukaryotes has lagged behind that on prokaryotes [4–7].

In agroecosystems, many microorganisms at the soil–water interface form microbial aggregates [8, 9]. Among these, periphytons—ubiquitous microbial aggregates in paddy fields—play critical roles in regulating element cycling, such as manganese cycling and ammonia volatilization, within paddy field ecosystems [10, 11]. Advances in periphyton research empower us to harness their ecological functions to improve rice productivity [12].

Periphyton communities are biologically diverse, comprising both prokaryotes and microeukaryotes [13, 14]. While the prokaryotic components of periphyton have been systematically studied [15], our understanding of the microeukaryotic components remains limited. To complement our current knowledge of periphyton in paddy fields, dedicated and integrated studies on microeukaryotes are now essential. A comprehensive understanding of periphyton will guide the development of strategies for regulating their growth and advancing periphyton-based biotechnologies for sustainable rice production.

The biodiversity within periphyton underpins its varied roles in paddy fields. Numerous studies have examined how nutrients and stoichiometry affect soil bacterial diversity [16, 17], highlighting the influence of nutrient availability (e.g. phosphorus) and stoichiometric ratios (carbon: nitrogen, nitrogen: phosphorus, and carbon: phosphorus) on soil bacterial diversity and composition across both natural and human-impacted ecosystems. However, less attention has been given to the effects of these factors on microeukaryotic diversity, especially within periphyton. Given the widespread anthropogenic disturbances that alter soil elemental stoichiometry in agroecosystems [16] and the recognized significance of periphytons as microbial nutrient sinks in paddy fields [13, 14], we hypothesize that variations in the nutrient sink potential within periphytons lead to imbalanced intracellular nutrient stoichiometry. This imbalance could serve as a feedback mechanism, becoming a primary factor influencing microbial community composition at both local and regional scales.

To test this hypothesis, we first assessed the carbon (C), nitrogen (N), and phosphorus (P) storage capacities of periphytons collected from paddy fields, along with their resulting intracellular nutrient stoichiometries. We then analyzed how these stoichiometries influence local species richness, regional variations in microeukaryotic communities, and the assembly of both abundant and rare taxa within periphytons. In summary, the following questions were addressed: (i) What are the diversity patterns and mechanisms that sustain microeukaryotic diversity in periphyton across different spatial scales? (ii) How do intracellular nutrient stoichiometries influence the spatial dynamics of microeukaryotic communities within periphyton?

## Materials and methods

### Sampling areas and collection

We collected periphytons from a total of 220 paddy fields located in 22 major rice planting areas, covering latitudes from 18°46′ N to 47°16′ N. These fields were strategically distributed across Northeast China (Tieling and Dandong in Liaoning Province; Wuchang and Qiqihar in Heilongjiang Province), Central China (Changshu and Yancheng in Jiangsu Province; Ningbo and Hangzhou in Zhejiang Province; Wuhu and Chizhou in Anhui Province; Yueyang in Hunan Province; Jingzhou, Yichang, and Wuhan in Hubei Province; Jiujiang and Yingtan in Jiangxi Province), and South China (Taishan and Renhua in Guangdong Province; Quanzhou, Fuzhou, and Nanping in Fujian Province; and Ledong in Hainan Province) (Table S1). From each paddy field, we collected 10 samples of periphyton, paddy soil, and floodwater, following the methods described in our previous studies [13, 14]. All samples were taken 7–15 days after transplanting. In total, 220 samples each of periphyton, paddy soil, and floodwater were gathered for this study.

### Intracellular nutrient and mineral elements detection

Soil and periphyton samples were placed in aluminum containers and dried in an oven at 60°C until reaching constant weights. The total organic carbon (TOC), total nitrogen (TN), and total phosphorus (TP) contents in both periphyton and soil were subsequently determined (Table S1). For TOC and TN analysis, the samples were treated with 0.1 M HCl to remove soil carbonate before being analyzed using a C/N analyzer (FLASH 2000 NC Analyzer, Thermo Scientific). The TP content in periphyton and soil samples was quantified using the acid oxidation method, involving wet digestion with HClO_4_ and H_2_SO_4_ [15, 18]. Mineral element contents (e.g. Fe, Mg) in periphyton were quantified using atomic absorption spectrometry following aqua regia digestion (Thermo Scientific™ iCE™ 3000, MA, USA).

### 18S rRNA gene amplicon sequencing and data analysis

To analyze the microeukaryotic microbial composition of periphytons from various sampling areas, 2 g of periphyton samples were taken from each of the 10 samples. These samples were thoroughly mixed and then divided into three equal parts. Total DNA was extracted from each periphyton sample (2 g, wet weight) using DNA extraction kits (MOBIO 12888-100, Carlsbad, CA, USA). The content and purity of the extracted DNA were measured using the NanoDrop One spectrophotometer (Thermo Fisher Scientific, MA, USA). Sequencing libraries were generated using the NEB Next Ultra DNA Library Prep Kit for Illumina (New England Biolabs, Beverly, MA, USA) according to the manufacturer’s instructions. Primers 528F (5′-GCGGTAATTCCAGCTCCAA-3′) and 706R (5′-AATCCRAGAATTTCACCTCT-3′) [19] were used for detecting microeukaryotic communities on the HiSeq 2500 platform, performed at Guangdong Magigene Biotechnology Co., Ltd. (Guangzhou, China). Quality filtering of the raw reads was performed under specific conditions using the Cutadapt (v1.9.1, http://cutadapt.readthedocs.io/en/stable/) quality control process to obtain high-quality clean reads. The reads were compared with the reference database (SILVA SSU and LSU databases 128) using the UCHIME algorithm to detect and remove chimera sequences [20].

Sequence analysis was performed using UPARSE software [21], where sequences with ≥97% similarity were assigned to the same operational taxonomic unit (OTU). Taxonomic information was annotated using the SILVA 138 database (https://www.arb-silva.de/). Microbial sequences were deposited in the NCBI database under the accession number PRJNA854289. The obtained OTUs were classified into abundant and rare OTUs. Rare OTUs were defined as those with relative abundance <0.01% in all samples, whereas OTUs with relative abundance >1% were classified as abundant. OTUs with relative abundances ranging from 0.01% to 1% were classified as moderate OTUs [22].

### Statistical analyses

#### Geo-distribution patterns

The LDG theory [23] was employed to evaluate the geographical distribution pattern of local species richness (α-diversity) of microeukaryotes (entire, abundant, and rare taxa) in periphytons. This analysis was conducted using the Shannon index data and R Studio software, utilizing the packages “ggplot2,” “MASS,” “mgcv,” and “splines” (Systat Software Inc., CA, USA). To assess the patterns of spatial community variation, the distance-decay relationship (DDR) was used [24]. This involved calculating Bray–Curtis similarity (based on the average of 100 bootstraps) of each subcommunity (entire, abundant, and rare taxa) against the geographic distances between sampling sites. Distance-decay curves were visualized using the same R Studio software and packages as described above.

### Community-level B*com* analysis

To calculate the B*com* values for each microeukaryotic OTU (entire, abundant, and rare) in periphytons, we used the “niche.width” function from the “spaa” package in R. Subsequently, we determined the community-level B-value as the average of the B*com* values for entire taxa present in a given community or sampling area [25]. The B*com* values for entire, abundant, and rare microeukaryotic subcommunities were then visualized and compared using bar plots.

### Neutral community model (NCM) analysis

To assess the role of stochastic processes in the assembly of microeukaryotic subcommunities in periphytons, we employed the NCM analysis. We predicted the relationship between the detection occupancy frequency of abundant, rare, and entire OTUs (including both abundant, moderate, and rare) and their relative abundance across all samples [7]. The 95% confidence intervals for each fitting statistic were calculated using bootstrapping with 1000 replicates. The parameter *Nm*, where *N* represents the community size and *m* denotes the immigration rate, was used to estimate dispersal between communities. *R*^2^ values were used to evaluate the model fit. All computations were performed in R using the “picante,” “ape,” “rlang,” “ecodist,” “vegan,” “agricolae,” and “tidyverse” packages [26].

### Phylogeny-based community metrics analysis

We calculated β-nearest taxon index (βNTI) values using R with the “picante,” “ape,” “rlang,” and “ggpubr” packages to assess the relative roles of stochastic and deterministic processes in shaping microeukaryotic subcommunity assembly in periphytons [27]. Briefly, βNTI values were interpreted as follows: |βNTI| > 2 indicated deterministic processes predominated in community assembly, while |βNTI| < 2 suggested stochastic processes dominated [12, 20]. We further categorized these processes into five ecological types based on βNTI and Bray–Curtis-based Raup–Crick Index (RCBray) values: heterogeneous selection (βNTI < −2), homogeneous selection (βNTI > +2), dispersal limitation (|βNTI| < 2 and RCBray > 0.95), homogenizing dispersal (|βNTI| < 2 and RCBray < −0.95), and undominated (|βNTI| < 2 and |RCBray| < 0.95) [28, 29]. By calculating the proportions of these five ecological types in the assembly processes of both the entire community and its rare subcommunity, we can assess their relative contributions to shaping the overall community structure and the assembly of its rare taxa.

### Mantel tests

We conducted Mantel tests to examine the impacts of intracellular nutrients (TOC, TN, and TP) and their stoichiometries (C/N, C/P, and N/P), as well as mineral contents (Ca, Mg, and Fe), on α-diversity (Shannon index), β-diversity (Bray–Curtis similarity), and assembly processes (βNTI values) of both entire and rare microeukaryotic communities within periphytons. Pairwise comparisons of each factor were depicted using a color gradient representing Spearman’s correlation coefficient. The width of the edges reflects Mantel’s *r* statistic for corresponding distance correlations, while the color of the edges indicates statistical significance based on 9999 permutations.

### Partial least squares path modeling (PLS-PM) analysis

We employed PLS-PM to analyze the direct and indirect effects of intracellular nutrient stoichiometry (C/N, C/P, and N/P) on α-diversity (Shannon index), while simultaneously exploring the direct and indirect effects of α-diversity on β-diversity (Bray–Curtis similarity) of both entire and rare microeukaryotic communities within periphytons. PLS-PM analysis was conducted using the “plspm” package in R Studio software [24], and the results were visualized using PowerPoint 2019 (Microsoft, Redmond, Washington, USA).

## Results

### Imbalance intracellular nutrient stoichiometries

**Figure 1:**
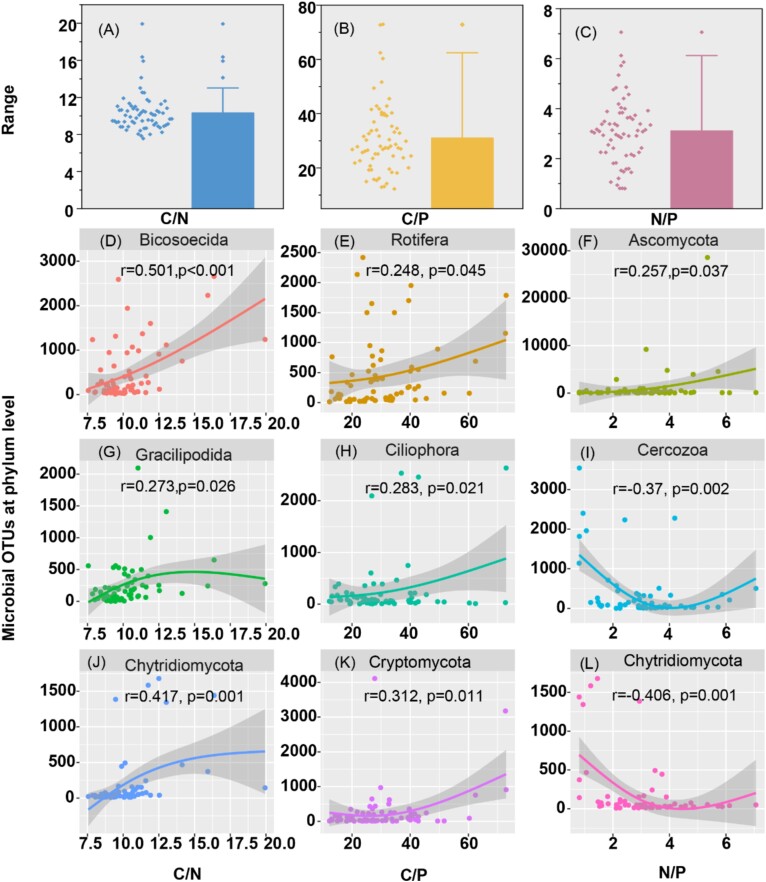
Intracellular nutrient stoichiometries and their correlation with microeukaryotic microbes within periphytons. Panels A-C display the results of carbon-to-nitrogen ratio (C/N), carbon-to-phosphorus ratio (C/P), and nitrogen-to-phosphorus ratio (N/P). Panels D, G, and J illustrate the OTUs of microeukaryotic microbes significantly associated with C/N, while panels E, H, and K, as well as panels F, I, and L, depict the OTUs of microeukaryotic microbes significantly associated with C/P and N/P, respectively (p < 0.05).

Periphytons exhibited remarkable variability in C, N, and P accumulation, characterized by high C, low N, and low P levels. This variability resulted in imbalanced intracellular nutrient stoichiometries across periphytons, particularly evident in the C/N ratio (average value = 10.3 ± 2.1, Fig. 1A), C/P ratio (average value = 30.9 ± 13.1, Fig. 1B), and N/P ratio (average value = 3.1 ± 1.3, Fig. 1C).

### Taxonomic compositions and relative abundances of microeukaryotes

Periphyton samples exhibited a diverse range of microeukaryotic species (Table S2). Rare OTUs comprised a substantial proportion of the total OTUs across all periphyton samples, varying from 76.41% in Yancheng to 88.24% in Yingtan. Despite their high representation, the relative abundances of these rare OTUs were relatively low, ranging from 3.66% in Ledong to 5.47% in Fuzhou. Conversely, abundant taxa constituted a smaller fraction of the OTUs, from 0.34% in Hangzhou to 0.70% in Renhua, but these taxa accounted for a remarkable proportion of the total relative abundance, ranging between 25.48% in Dandong and 75.89% in Ledong. Moreover, moderate taxa represented a considerable segment of the OTUs, from 11.29% in Yingtan to 22.23% in Yancheng, and their relative abundance ranged from 20.45% in Changshu to 69.15% in Yancheng (Table S2).

### Correlation between imbalanced nutrient stoichiometries and microeukaryotic growth

Microeukaryotic taxa at the phylum level that showed significant correlations with nutrient stoichiometries are presented in Fig. 1D–I (*P* < .05). Elevated C/N ratios positively correlated with the abundance of *Bicosoecida* (*r* = 0.501, *P* < .001, Fig. 1D), *Gracilipodida* (*r* = 0.273, *P* = .026, Fig. 1G), and *Chytridiomycota* (*r* = 0.417, *P* = .001, Fig. 1J) in periphytons. Higher C/P ratios tended to favor *Rotifera* (*r* = 0.248, *P* = .045, Fig. 1E), *Ciliophora* (*r* = 0.283, *P* = .0215, Fig. 1H), and *Cryptomycota* (*r* = 0.312, *P* = .011, Fig. 1K). Conversely, higher N/P ratios correlated positively with *Ascomycota* (*r* = 0.257, *P* = .037, Fig. 1F) but negatively with *Cercozoa* (*r* = −0.37, *P* = .002, Fig. 1I) and *Chytridiomycota* (*r* = −0.406, *P* = .001, Fig. 1I).

### Geo-distribution patterns of the microeukaryotes across China

**Figure 2:**
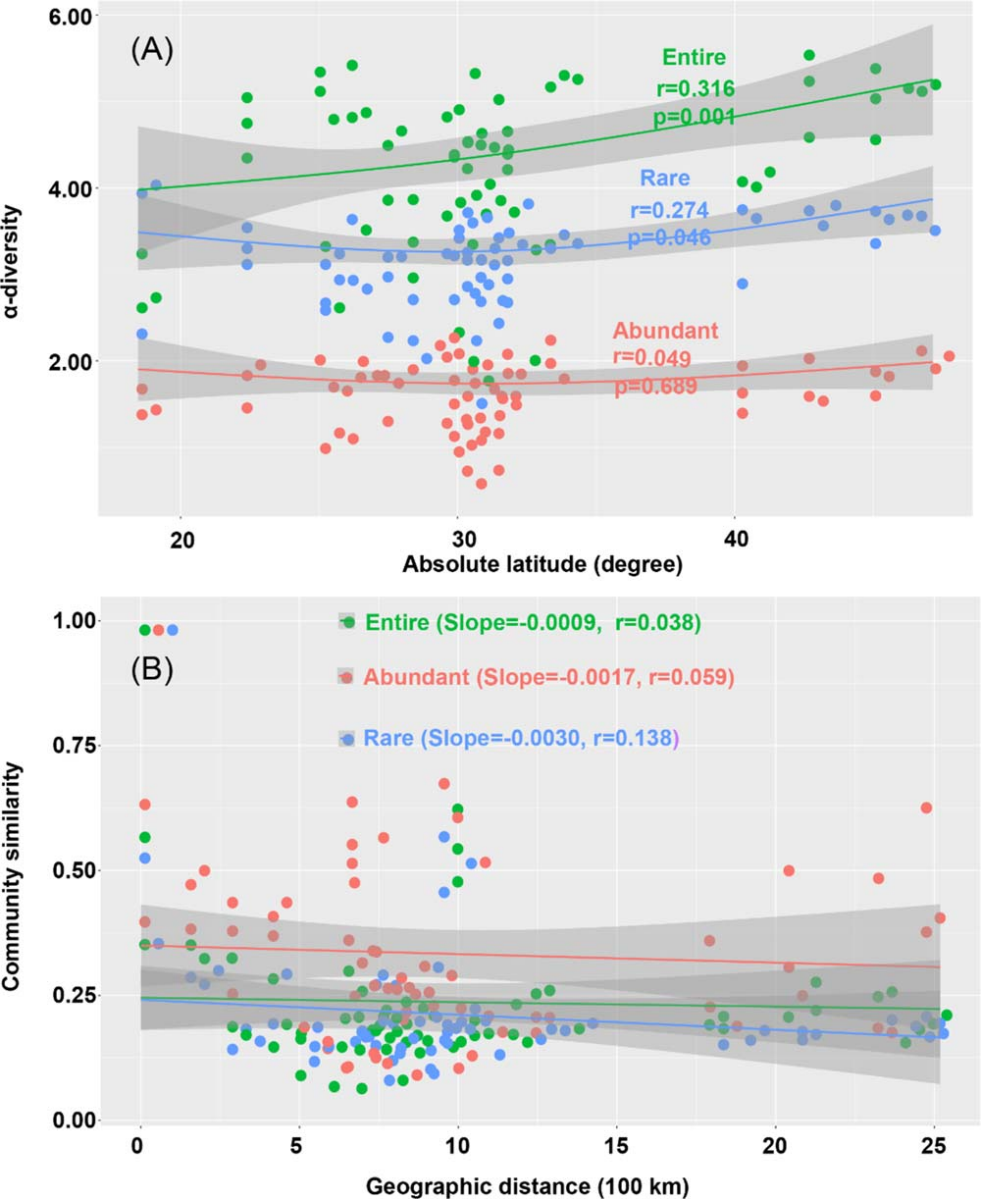
Geo-patterns of α-diversity and β-diversity microeukaryotic subcommunities in periphytons across China. Panel A: Geo-distribution patterns of α-diversity (Shannon index) of the entire, abundant and rare subcommunities along with the latitudes of sampling sites in China. Panel B: Distance-decay curves showing Bray–Curtis similarity against geographic distances between sampling sites. Solid lines denote the ordinary least-squares linear regressions.

Contrary to the LDG theory, which suggests that species richness generally increases with proximity to the equator [30], we observed an increase in α-diversity with latitude for both entire (*r* = 0.316, *P* = .001, Fig. 2A) and rare (*r* = 0.274, *P* = .046, Fig. 2A) microeukaryotic communities. However, the abundant subcommunity showed no significant correlation with latitude (*r* = 0.049, *P* = .689, Fig. 2A). DDRs indicated weak similarity decay among entire, abundant, and rare microeukaryotic subcommunities over a maximum geographic distance of 3469 km (Fig. 2B), suggesting non-ubiquitous distributions across China.

### Community assembly dynamics and environmental adaptability

**Figure 3:**
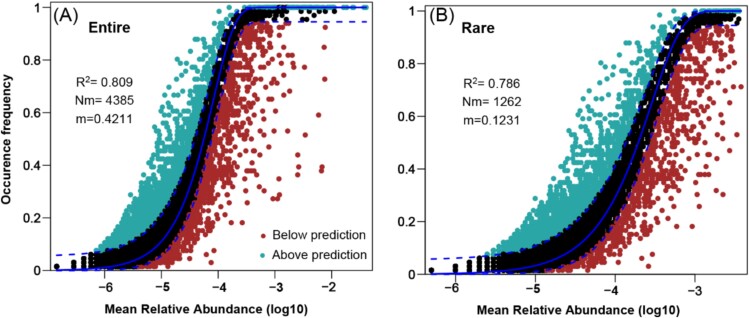
Fit of the neutral community model (NCM) of the entire (A) and rare (B) subcommunities assembly. The solid lines indicate the best fit to the NCM, and the dashed lines represent 95% confidence intervals around the model prediction. *Nm* indicates the metacommunity size times immigration, *N* represent the size of detected community, *m* shows the immigration rate, and *R*^2^ indicates the fitness to this model.

NCM effectively assessed the relationship between occurrence frequency and relative abundance variations of entire and rare OTUs, explaining 80.9% (Fig. 3A) and 78.6% (Fig. 3B) of community variance, respectively. These findings highlight the interplay of stochastic and deterministic processes in shaping microeukaryotic subcommunity assembly, with stochastic processes predominating. Conversely, NCM’s inability to estimate these relationships for abundant OTUs suggests their wider distribution across habitats. The calculated *Nm* values for the entire and rare subcommunities were 4385 and 1262, respectively, accompanied by estimated *m* values of 0.42 and 0.12 (Fig. 3). These findings suggest that the entire community experiences greater species limitation, more pronounced stochastic processes, and exhibits higher migration capacity compared to the rare subcommunity.

**Figure 4:**
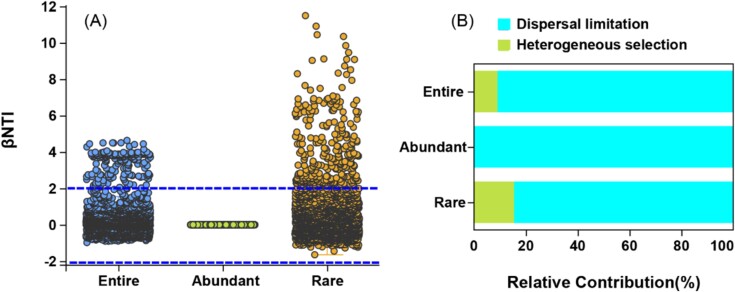
Results of phylogeny-based community metrics analysis of the entire, abundant, and rare subcommunities. Panel A: the βNTI values of each subcommunities, and panel B: the relative contribution of dispersal limitation and heterogeneous selection on the subcommunities assembly.

Quantifying the contribution of stochastic versus deterministic processes using βNTI values (Fig. 4), we observed that 91.0%, 100%, and 85.1% of the βNTI values for entire, abundant, and rare subcommunities fell within |βNTI| < 2. This indicates the predominant influence of stochastic processes on abundant subcommunities, particularly through dispersal limitation. Both deterministic and stochastic processes shaped the assembly of entire and rare communities, with deterministic processes contributing more remarkably to rare subcommunities than to the entire community (Fig. 4). Heterogeneous selection exerted a greater impact on rare subcommunity assembly (15.4%) compared to the entire community (9.0%), while dispersal limitation strongly affected abundant subcommunities (100%) relative to entire (91.0%) and rare subcommunities (84.5%).

**Figure 5:**
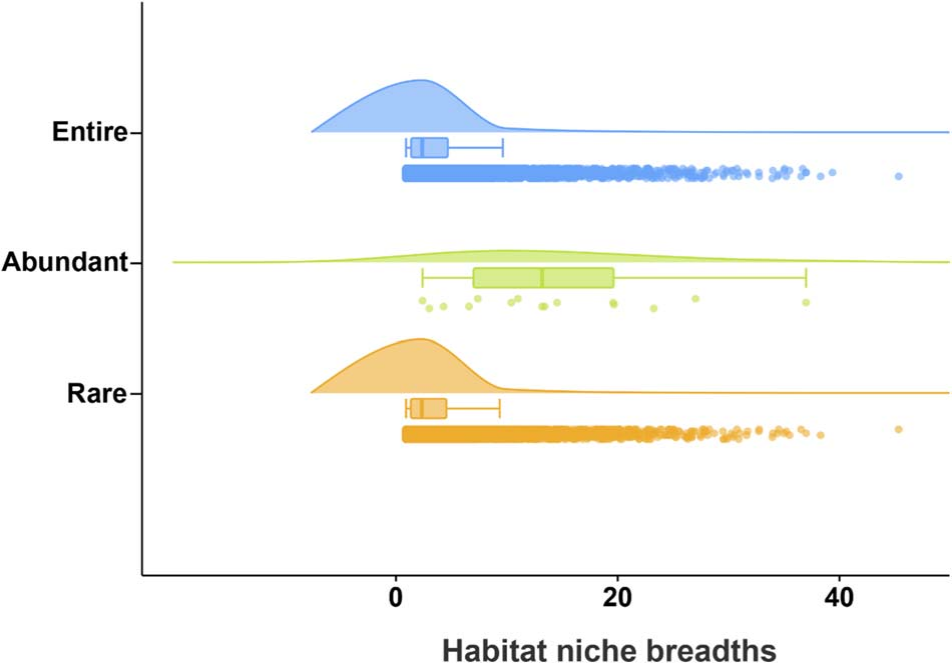
Results of subcommunity-level habitat niche breadths (*Bcom*) analysis. In the figures, boxplots and the nuclear density curve shown by the cloud plot were used to display the distribution status of original data. Jitter scatters represent the dispersion degree of data.

Additionally, B*com* values were computed to assess environmental adaptability among microeukaryotic subcommunities (Fig. 5). Abundant subcommunities exhibited a higher mean B*com* value (14.27 ± 0.01) compared to entire (8.42 ± 0.71) and rare subcommunities (8.19 ± 0.44). These results underscore the complex interplay of environmental filtering, species interactions, and dispersal dynamics in shaping the structure and adaptability of microeukaryotic communities within periphytons.

### Imbalance intracellular nutrient stoichiometries affected the local and spatial community diversity

**Figure 6:**
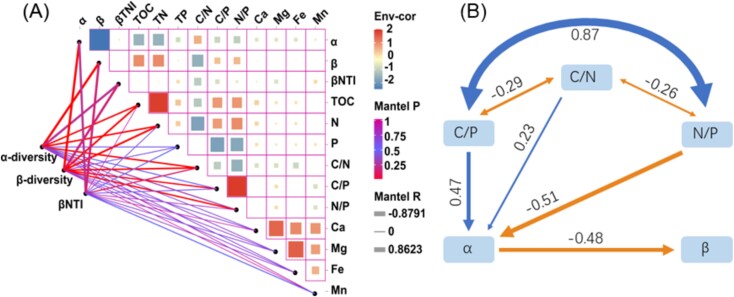
Effect of mineral elements (Ca, Mg, Fe, and Mg), nutrients (TOC, TN, and TP) and the imbalanced stoichiometries (C/N, C/P, and N/P) on the diversity of the microeukaryotic subcommunities. Panel A: Mantel tests results showed the correlation of mineral elements, nutrients and their stoichiometry on the assembly and diversity of the microeukaryotic subcommunities; panel B: PLS-PM results show the effects of nutrients stoichiometries on the α-diversity (Shannon index), and then the effects of α-diversity on the β-diversity (Bray–Curtis similarity) of the microeukaryotic communities in periphytons. Red lines present negative effect; blue lines present positive effect. The thickness of each line is proportional to the size of the effect.

Elevated levels of C and P, coupled with reduced N content in periphyton, constrain local species richness. Conversely, higher C and P levels, along with lower N content, enhance community diversity. As demonstrated in the Mantel test results (Fig. 6A), α-diversity within the entire community shows significant correlations with C/N (*r* = 0.43, *P* < .001) and N/P (*r* = −0.26, *P* = .03), while β-diversity correlates significantly with C/N (*r* = −0.48, *P* < .001), C/P (*r* = 0.35, *P* = .004), and N/P (*r* = 0.38, *P* = .001). Additionally, βNTI values are notably correlated with α-diversity (*r* = 0.82, *P* = .03), β-diversity (*r* = 0.86, *P* = .02), and C/N (*r* = −0.29, *P* = .02), indicating the significant association between imbalanced nutrient stoichiometries and diversity metrics.

Moreover, as illustrated in the PLS-PM results (Fig. 6B), imbalanced intracellular nutrient stoichiometries, particularly C/N (path coefficient = 0.23) and C/P (path coefficient = 0.47), positively influence the local species richness of microeukaryotes, whereas N/P exerts a large negative effect (path coefficient = −0.51). Similarly, C/N (path coefficient = 0.36) and C/P (path coefficient = 0.02) positively impact α-diversity, while N/P again demonstrates a significant negative effect (path coefficient = −0.51, *P* = .025). These findings underscore the diverse effects of imbalanced nutrient stoichiometries on local species richness.

Finally, under the influence of intracellular nutrient stoichiometries, local species richness of microeukaryotes plays a crucial role in shaping spatial community variations (Fig. 6B). This influence occurs through both direct (path coefficient = −0.48) and indirect (path coefficient = −0.03) pathways, highlighting the pivotal role of imbalanced nutrient stoichiometries as environmental filters.

## Discussion

Recent years have witnessed rapid advancements in the study of diversity, assembly, and biogeography of prokaryotes in natural ecosystems [4–7]. However, there remains a critical gap in large-scale investigations focusing on the microeukaryotic community within agroecosystems [20, 31], particularly in habitats like periphyton, which thrives at the soil–water interface in paddy fields. Our study addresses this gap by providing a comprehensive analysis of nutrient accumulation potentials and resulting intracellular nutrient stoichiometries, and their profound impacts on the structure, biogeography, and assembly processes of microeukaryotic taxa in periphytons. We found that imbalanced intracellular nutrient stoichiometries remarkably influence the local species richness of microeukaryotic communities within periphytons, thereby dictating their spatial community variation. This novel understanding highlights the complex interplay between nutrient dynamics and microbial community dynamics in agroecosystems.

### Diversity and distinct geo-distribution patterns of the microeukaryotic subcommunities

Although periphyton harbors a diverse array of microeukaryotic species, their diversity is notably lower compared to its prokaryotic counterparts [15]. Specifically, the number of OTUs and their proportions differ remarkably. Rare prokaryotic taxa constitute 43.7% of the total relative abundance within the prokaryotic community [15], whereas rare microeukaryotic taxa have a much lower relative abundance, with the highest recorded at 5.47% (c.f. Table S2). In contrast, abundant microeukaryotic taxa contribute a larger share of the relative abundance compared to their prokaryotic counterparts [15].

Unlike prokaryotic communities, where the entire prokaryotic community, as well as the moderate and rare subcommunities, conform to the typical LDG theory [15], microeukaryotes in periphytons demonstrate relatively low α-diversity and exhibit unique spatial distribution patterns that diverge from conventional latitudinal diversity gradients observed in prokaryotes (c.f. Fig. 2). This suggests that traditional ecological theories, such as the metabolic theory of ecology, may inadequately explain the diversity and distribution of microeukaryotes in these ecosystems [32]. We propose that these differences arise from the higher trophic level and niche specificity of microeukaryotic microorganisms, which feed on prokaryotic counterparts, as outlined by the food chain theory [33, 34].

### Microeukaryoic community assembly dominated by two processes

Quantitative assessment of community assembly processes reveals that stochastic processes, particularly dispersal limitation, dominate the assembly of abundant microeukaryotic subcommunities, whereas both stochastic and deterministic processes shape the assembly of entire and rare subcommunities (Figs 3 and 4). This highlights the importance of niche breadth, where organisms with wider niches exhibit greater metabolic flexibility and resilience [35]. Our findings support the notion that abundant microeukaryotic taxa, characterized by wider habitat tolerances (as indicated by higher B*com* values), are less influenced by environmental factors compared to entire and rare subcommunities (Fig. 5).

Furthermore, employing the NCM underscores the crucial role of stochastic processes, such as dispersal and ecological drift, in shaping microeukaryotic community assembly [25, 36]. The inability of NCM to accurately model the abundant subcommunity suggests that these taxa exhibit broader dispersal capabilities compared to rare taxa, corroborating previous studies highlighting dispersal limitation as a key factor driving community structure [37]. Null model analysis further supports our findings by providing insights into the relative contributions of deterministic and stochastic processes in community assembly.

### Imbalanced intracellular nutrient stoichiometry-triggered local species richness dictates the spatial community variation

Recent research focusing on nutrient stoichiometry and its impact on soil bacterial diversity has underscored the critical role of soil nutrient ratios compared to other environmental factors, such as climate, soil pH, and root influences [17, 38]. However, the influence of imbalanced intracellular nutrient stoichiometries on local species richness and spatial community variation in periphytons across paddy fields remains poorly understood. Here, we identify imbalanced intracellular nutrient stoichiometries as crucial environmental filters that influence local species richness and spatial variability of microeukaryotic communities within periphytons (Fig. 6). Periphytons serve as reservoirs for accumulating substantial amounts of C, N, and P [39], where intracellular nutrient availability plays a pivotal role in shaping diversity and community assembly dynamics compared to soil nutrients.

Furthermore, our findings highlight that high levels of C and P and low N content in periphytons constrain local species richness while promoting community diversity. The influence of nutrient stoichiometry on α-diversity likely cascades to impact β-diversity patterns among microeukaryotic communities. Nutrient enrichment alters microbial community composition by favoring the growth of specific taxa, a phenomenon supported by the growth-rate hypothesis suggesting that fast-growing organisms like bacteria exhibit high demand for P and possess a low C [40]. However, this hypothesis may not fully apply to microeukaryotic communities, which exhibit relatively slower growth rates. Additionally, while C and N are readily obtained from the atmosphere by periphytons through photosynthesis and N fixation [13], P availability predominantly regulates community assembly and diversity in periphytons [17]. Thus, limited P supply emerges as a critical factor restricting microeukaryotic diversity. Periphytons with high P content and low C ratios support greater microbial diversity (Fig. 6), whereas those with low P content and high C ratios limit community assembly through competitive exclusion among microbial species [17, 41].

## Conclusion

In summary, our study elucidates how periphytons sequester nutrients and how variations in nutrient accumulation potential lead to imbalanced intracellular nutrient stoichiometries, thereby shaping the geographical imprint of microeukaryotic communities in paddy field ecosystems. Notably, the spatial patterns of local microeukaryotic species richness do not conform to the conventional latitudinal diversity gradient theory but rather align with the distance-decay model. This research enhances our understanding of microeukaryotic communities within periphytons, emphasizing the intricate interplay between microbial aggregates and nutrient dynamics. Ultimately, these insights contribute to a broader comprehension of the factors influencing regional structural variations of microeukaryotes at the soil–water interface in agricultural landscapes.

## Supplementary Material

Supplemental_Material_ycae119

## Data Availability

All raw sequences from this study have been submitted to the NCBI Sequence Read Archive database under the BioProject number PRJNA854289.
