# Supplementary material for: Imbalanced intracellular nutrient stoichiometries drive the regional structural variation of microeukaryotic communities in paddy fields
Source: ISME Commun. 2024 Oct 10;4(1):ycae119. doi: 10.1093/ismeco/ycae119 (PMC11512751; doi:10.1093/ismeco/ycae119)
Supplement: Supplemental_Material_ycae119 [file supplemental_material_ycae119.docx]

Table S1 Geographic location of each sampling site and its corresponding soil characteristics.

| Site | Longitude | Latitude | TOC (g/kg) | TN (g/kg) | TP (g/kg) |
| --- | --- | --- | --- | --- | --- |
| Ledong | 18.46 | 108.88 | 21.89±5.73 | 2.11±0.28 | 0.64±0.05 |
| Taishan | 22.25 | 112.79 | 28.35±6.10 | 2.59±0.53 | 0.94±0.17 |
| Renhua | 25.09 | 113.75 | 18.27±2.01 | 1.57±0.09 | 0.53±0.03 |
| Quanzhou | 24.89 | 118.61 | 10.97±3.73 | 0.89±0.39 | 0.95±0.55 |
| Fuzhou | 26.05 | 119.27 | 13.32±1.59 | 1.16±0.13 | 0.69±0.08 |
| Nanping | 27.33 | 118.12 | 13.89±2.95 | 1.22±0.28 | 0.41±0.12 |
| Changshu | 31.65 | 120.75 | 18.66±3.41 | 1.73±0.33 | 0.74±0.12 |
| Yancheng | 33.20 | 120.50 | 17.10±1.89 | 1.91±0.24 | 0.73±0.02 |
| Ningbo | 29.90 | 121.84 | 28.17±3.16 | 2.94±0.27 | 0.96±0.09 |
| Hangzhou | 30.21 | 120.21 | 34.51±6.50 | 3.15±0.54 | 0.67±0.13 |
| Wuhu | 31.34 | 118.39 | 32.80±5.85 | 3.07±0.56 | 0.90±0.11 |
| Chizhou | 30.69 | 117.57 | 23.15±7.41 | 2.19±0.56 | 0.43±0.07 |
| Yueyang | 29.46 | 113.01 | 23.21±0.88 | 2.48±0.07 | 0.52±0.05 |
| Jingzhou | 30.35 | 112.19 | 16.87±3.71 | 1.66±0.28 | 1.13±0.11 |
| Yichang | 30.53 | 111.43 | 15.98±2.66 | 1.54±0.28 | 0.51±0.08 |
| Wuhan | 30.48 | 114.32 | 23.71±3.57 | 2.20±0.34 | 1.32±1.01 |
| Jiujiang | 29.73 | 115.99 | 24.49±6.03 | 2.16±0.44 | 0.49±0.17 |
| Yingtan | 28.24 | 117.04 | 19.32±4.58 | 1.67±0.43 | 0.42±0.07 |
| Tieling | 42.55 | 124.16 | 19.25±2.58 | 1.58±0.17 | 0.60±0.04 |
| Dandong | 40.14 | 124.40 | 12.97±3.13 | 1.14±0.25 | 0.72±0.06 |
| Wuchang | 44.93 | 127.17 | 27.36±6.36 | 1.84±0.29 | 0.74±0.07 |
| Qiqihar | 47.16 | 123.82 | 23.61±3.03 | 1.82±0.22 | 0.73±0.07 |

Table S2 Taxonomic compositions and relative abundances of abundant, moderate, and rare taxa of microeukaryotes in periphyton samples

| Sites | OTUs number | | | | | Relative abundance (%) | | |
| --- | --- | --- | --- | --- | --- | --- | --- | --- |
|  | Entire | Abundant | | Moderate | Rare | Abundant | Moderate | Rare |
| Quanzhou | 3441 | | 17 | 683 | 2741 | 35.55 | 59.44 | 5.01 |
| Wuhu | 2881 | | 10 | 493 | 2378 | 52.94 | 42.85 | 4.20 |
| Wuchang | 3197 | | 19 | 642 | 2536 | 42.33 | 52.72 | 4.94 |
| Renhua | 2421 | | 17 | 408 | 1996 | 71.36 | 24.79 | 3.86 |
| Jingzhou | 3290 | | 16 | 615 | 2659 | 51.41 | 43.53 | 5.06 |
| Chizhou | 2899 | | 20 | 496 | 2383 | 51.09 | 44.76 | 4.15 |
| Hangzhou | 2664 | | 9 | 357 | 2298 | 70.09 | 25.71 | 4.20 |
| Jiujiang | 3127 | | 17 | 475 | 2635 | 56.84 | 38.80 | 4.36 |
| Yingtan | 3027 | | 14 | 342 | 2671 | 72.34 | 23.60 | 4.06 |
| Nanping | 3014 | | 14 | 617 | 2383 | 47.07 | 48.11 | 4.81 |
| Fuzhou | 3686 | | 18 | 686 | 2982 | 39.76 | 54.77 | 5.47 |
| Ningbo | 2862 | | 18 | 472 | 2372 | 55.62 | 39.85 | 4.53 |
| Yueyang | 3301 | | 13 | 540 | 2748 | 56.87 | 38.06 | 5.06 |
| Taishan | 3054 | | 21 | 597 | 2436 | 50.38 | 44.59 | 5.03 |
| Yichang | 3104 | | 13 | 455 | 2636 | 61.11 | 34.17 | 4.71 |
| Changshu | 3167 | | 14 | 599 | 2554 | 49.37 | 46.01 | 4.62 |
| Ledong | 1644 | | 9 | 354 | 1281 | 75.89 | 20.45 | 3.66 |
| Tieling | 3193 | | 16 | 726 | 2451 | 36.07 | 58.81 | 5.12 |
| Qiqihar | 2996 | | 14 | 513 | 2469 | 53.51 | 42.21 | 4.29 |
| Dandong | 3268 | | 20 | 724 | 2524 | 35.88 | 58.79 | 5.33 |
| Yancheng | 3353 | | 12 | 779 | 2562 | 25.48 | 69.15 | 5.38 |
| Wuhan | 3094 | | 17 | 593 | 2484 | 45.54 | 49.75 | 4.71 |
